# The Catalytic Effect of Iron and Alkali and Alkaline Earth Metal Sulfates Loading Series on the Conversion of Cellulose-Derived Hydrochars and Chars

**DOI:** 10.1021/acsomega.3c00887

**Published:** 2023-03-10

**Authors:** Till Eckhard, Christin Pflieger, Jannik Böttger, Pascal Telaar, Francesca Cerciello, Martin Muhler

**Affiliations:** Laboratory of Industrial Chemistry, Ruhr University Bochum, 44780 Bochum, Germany

## Abstract

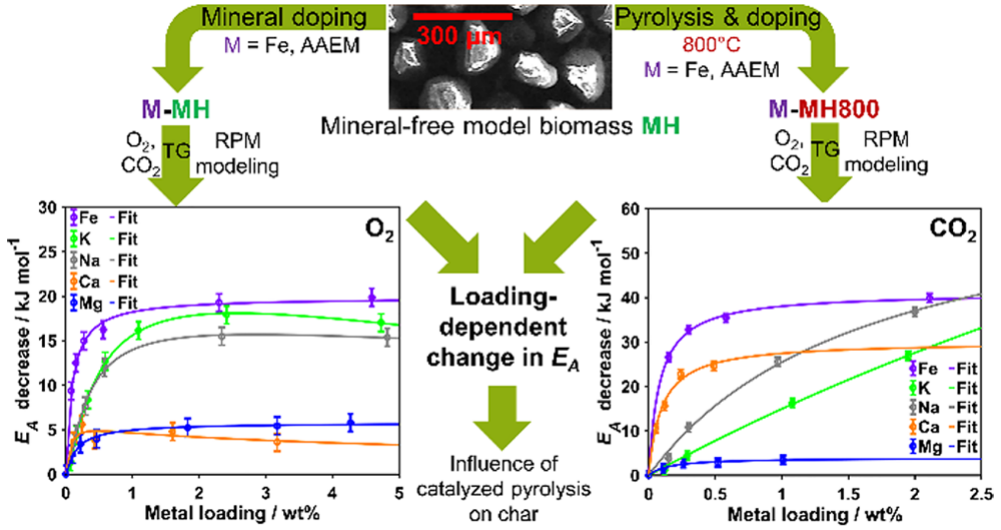

The catalytic effect of minerals on biomass conversion was studied focusing on Fe as well as alkali and alkaline earth metals as the metallic inorganic elements typically present in minerals found in biomass. A mineral-free reference hydrochar and an analogous char material based on cellulose were systematically doped with sulfates of the different metallic inorganic elements in various amounts via impregnation, thereby excluding differences originating from the counterion and the carbon matrix. Thermogravimetric reactivity measurements were performed in diluted O_2_ and CO_2_, and the derivative thermogravimetry curves were fitted using the random pore model. This procedure enabled a quantification of the apparent activation energy decrease due to doping as well as the influence of doping on the carbon structural parameter. Fe sulfate was always among the most active minerals, and alkali metal sulfates were typically more active than alkaline earth metal sulfates. The only exception was the high activity of very small Ca sulfate loadings during gasification. A saturation behavior of the kinetic parameter upon increasing the mineral loading was observed. The Langmuir-type modeling of this dependence further revealed that catalytically influenced devolatilization results in a char with higher oxidation reactivity, whereas for gasification, thermal annealing dominates. The systematically derived parameters provide a comprehensive description of catalytic effects, taking into account the type of mineral, the applied loading, the used atmosphere, and the fuel morphology. The derived activation energies can be used to include catalytic effects into combustion models.

## 1 Introduction

Biomass oxy-fuel combustion is a promising technique that may contribute to the mitigation of global warming by reducing anthropogenic greenhouse gas emissions.^1−3^ Modeling of oxy-fuel combustion consisting of pyrolysis and char conversion is used for the improvement of this high potential technology.^1^ However, additional challenges can arise from the different minor constituents in the wide range of biomass sources.^4^ Special focus lies on minerals which may alter the combustion behavior of biomass significantly.^5^ Catalytic activity was found for minerals of alkali and alkaline earth metals (AAEM) as well as of the transition metal Fe in different oxy-fuel-related conversion atmospheres.^6−8^ Different mineral phases such as oxides, carbonates, chlorides, and sulfates may be present. The latter represent important species during the conversion of biomass, as they are formed from original organic and inorganic matter before being transformed to more stable oxides.^9^ A number of studies have investigated the catalytic effects of metallic inorganic elements, however, focusing on different types of carbon material and minerals.^10−15^ Hence, the comparability of reactivities derived in these studies is limited, as investigated effects depend on the fuel type as well as both the cations and anions present in the minerals. Typically, reported trends were referred to differences of the metallic inorganic elements, but this assignment is only reliable when using consistent types of biomass and minerals.

In general, the catalytic activity of minerals depends on the applied temperature,^16^ the loading amount,^15,17−21^ the counterions and/or other mineral phases,^18,22,23^ and the biomass-component ratio.^17^ The focus of many studies was the sustainable valorization of biomass to biogas, bio-oil, and biochar,^24,25^ whereas systematic research on the catalytic influences on biomass in combustion excluding intrinsic differences between varied biomass, heating rates, or used catalysts is still limited.^23,26^ Thus, a generalized description and especially quantification of catalytic effects is impeded by differences in the morphology,^27^ in the mineral content and type, and in the degree of contact between the mineral and the carbon structure.^28,29^

During char conversion, K-containing minerals were typically reported to exhibit the highest activity, whereas the reactivity order of minerals containing Na, Ca, Mg, and Fe was controversial. For example, the reactivity of fir tree sawdust char gasification was improved by the doping of a mixture of acetates and nitrates in the order K > Na > Ca > Fe > Mg referring to the metallic inorganic elements present in the doped minerals.^30^ In comparison, pistachio nutshell gasification using nitrates was improved in the order Na > Ca > Fe > K > Mg.^31^ In addition to these comparative studies deriving a differently pronounced impact of metallic inorganic elements in the form of reactivity orders,^10,30^ more specific quantifications of catalytic effects were obtained in recent years by loading series of selected minerals.^11,15,32,33^ In this way, activation energies were correlated to defined amounts of certain minerals, but these investigations focused only on a specific type of dopant. For example, the loading of different K salts on wheat straw was found to result in an activation energy decrease of up to 59% in char oxidation,^11^ while for CO_2_ gasification of sawdust char different alkali salts were reported to lower the activation energy by even up to 72%.^14,33^ In the same atmosphere, less pronounced activation energy decreases resulted from AAEM acetates doped on pine char (53%) and especially, from FeCl_3_ doping on sawdust char (11%).^10,32^ It was discussed whether FeCl_3_ loading affects char reactivity only through catalytic activity or in combination with changes of the carbonaceous structure due to increased graphitization and carbon structural ordering. CaO was reported to be an especially good catalyst for gasification producing more H_2_ and less CO_*x*_^34^ based on its strong promotion of the water–gas shift reaction by CO_2_ sorption. The description of the effects of Mg-containing minerals is the most complex. They were reported to have insignificant or no activity in H_2_O gasification,^31^ whereas in CO_2_ gasification even a promoting role of Mg compounds in the deactivation of other metallic inorganic element species was indicated, presumably by forming inactive mixed metal minerals or by decreasing the contact between the catalytically active species and the carbon matrix.^35^ Typically, the catalytic activity is related to oxygen-transfer mechanisms in which the mineral phase traverses a reduction–oxidation (RedOx) cycle^11,36^ or to their ability to promote oxygen chemisorption based on different oxygenated intermediate species.^37−39^

Of further importance for the combustion process is the diffusion of gaseous reactant to the active sites.^40^ In addition to their catalytic effect, minerals were often reported to have also a structural influence on the conversion process.^41−44^ The blockage of carbon pores by the formation of mineral agglomerates and salt deposits during pyrolysis and/or char conversion, thereby hindering the access to the active sites in the char, was found for different gaseous reactants.^6,12,14^ Although first attempts on quantifying structural effects were made,^10^ further investigations on the role of minerals on conversion reactivity as a function of mineral type and amount are required also including the effect of (catalyzed) pyrolysis on the char reactivity.^6,7^

Currently, models do not account for the catalytic effects of minerals impeding the model-based retrofitting of already existing power plants.^4,45^ To contribute to a future quantification of mineral effects on the oxy-fuel combustion process, this work analyzes the individual effects of dopants on inherently mineral-free, cellulose-derived biomass model fuels by impregnation before and after devolatilization. In this way, not only the direct determination of the effect on char conversion is possible but also the effect of catalyzed pyrolysis on the generated char is obtained indirectly by fuel comparison. Relating to oxy-fuel atmospheres, investigations were performed in both diluted O_2_ and CO_2_. The derived kinetic parameters finally enable the implementation of catalytic effects based on the metallic inorganic element content of biomass fuels into common combustion models, such as the carbon burnout kinetics model (CCK/G)^46,47^ describing char combustion or the seamless CRECK-S-B model^48^ describing both pyrolysis and char conversion.

In a previous work, it was shown that there is a high agreement of apparent activation energies derived from applying kinetic modeling as well as adapting the more comprehensive CCK/G and CRECK-S-B models to thermogravimetric data.^49^ Within the heterogeneous reaction mechanisms comprising seven steps for CCK/G and four steps for CRECK-S-B, it was demonstrated that the decrease in activation energy of an individual step per each reactive gas enabled to account for the catalytic effect of minerals in the different oxy-fuel-related atmospheres. Consequently, an inclusion of kinetic effects into these two combustion models is possible by using the results of the less complex kinetic modeling to adapt the activation energies of decisive steps. Based on this approach, the kinetic parameters derived in the present work provide the data set to directly extend both the CCK/G and the CRECK-S-B for the effects of minerals containing different metallic inorganic elements in various amounts on the conversion of char, and in this way paving the way toward a more comprehensive predictability of biomass oxy-fuel combustion.

## 2 Materials and Methods

### 2.1 Materials

The loading series investigated in this work is obtained by selectively doping varied amounts of minerals on two mineral-free reference materials synthesized from the biomass component cellulose. Initially, the undoped hydrochar labeled ‘MH’ as reference material prior to devolatilization was derived by hydrothermal carbonization of microcrystalline cellulose spheres as described in refs (5) and (50). The reference material after devolatilization was the analogous char labeled ‘MH800’, subsequently obtained by the low heating rate pyrolysis of MH at 800 °C according to ref (49). MH and MH800 were chosen as mineral-free reference fuels because they are already well characterized enabling the easy identification of catalytic effects.^5,49−52^

Both undoped starting materials were then doped by impregnation using systematically varied amounts of different metal sulfates, namely, FeSO_4_·7H_2_O, K_2_SO_4_, Na_2_SO_4_, CaSO_4_·2H_2_O, and MgSO_4_. The amount of mineral used was adapted to reach a certain weight fraction of the contained metallic inorganic element relative to the char material. Initially, samples with 0.15 and 0.3 wt % were synthesized for all metallic inorganic elements, while additional loadings were selected individually based on first results of the initial doped samples. The doping procedure, as described in detail in ref (5), resulted in doped hydrochars and chars labeled according to the attempted weight fraction *w* and type of metallic inorganic element *M* as ‘*w M*-MH’ for doped hydrochars and ‘*w M*-MH800’ for doped chars, respectively. For example, 0.15 wt % FeSO_4_ doped on the char was denoted as ‘0.15 Fe-MH800’. The actual amount of metallic inorganic elements contained in the doped samples was determined from atomic absorption spectrometry (AAS) by Mikroanalytisches Laboratorium Kolbe.

A characterization of the reference materials as well as the determination of metallic inorganic element content in the doped samples as a validation of the impregnation procedure are provided in section S1. The analysis in section 3 focuses on the differences in reactivity of the doped samples, individually evaluating the influence of metallic inorganic element type and amount as well as of the fuel affected by the minerals. For an improved readability, descriptions refer to the metallic inorganic element of the sulfates impregnated on the carbon materials. The kinetic studies focus on the catalytic effects throughout the progress of oxidation and gasification. As all minerals contain the same anion, differences are only related to the type of metallic inorganic element, denoted by its element symbol despite being present as metal cation.

### 2.2 Experimental Procedure

The reactivities of the samples were determined by performing thermogravimetric (TG) experiments in a magnetic suspension balance.^49^ For temperature-programmed (TP) experiments in both reacting gases, about 30 mg of sample was placed in a quartz crucible which was then lowered into the furnace and flushed for 70 min with 100 mL min^–1^ of the selected reactive atmosphere. For oxidation experiments, 20% O_2_ (99.998% purity) in He (99.999% purity) were adjusted, whereas gasification was performed in 50% CO_2_ (99.998% purity) in He (99.999% purity). After flushing, the experiments were performed by heating with 5 °C min^–1^ to 800 °C in case of oxidation or by heating with 1 °C min^–1^ to 1100 °C in case of gasification. In the latter case, the lower heating rate was chosen as 1100 °C was the temperature limit of the balance and only by slow heating the gasification reaction occurred completely in this measurable range. For isothermal validation measurements, also 30 mg of sample was heated with 10 °C min^–1^ in 100 mL min^–1^ He to the different selected temperatures corresponding to the rising branch of the DTG signal measured in TP measurements of the respective sample. After reaching the desired temperature, the atmosphere was switched to a reactive atmosphere consisting of different O_2_/He or CO_2_/He mole fractions and held for 3 h. For oxidation, a temperature range of 300–450 °C was investigated, while for gasification temperatures between 700 and 1000 °C were studied.

### 2.3 Analytical Methods

#### 2.3.1 Modeling of Conversion Curves

The obtained TG data were qualitatively analyzed based on their differential, the DTG curves. For kinetic modeling, the obtained mass loss data were converted to time-dependent conversion *X*(*t*) data applying eq 1 with residual sample mass at a certain point in time *m*(*t*), initial mass *m*_0_, and mass of remaining ash *m*_*ash*_.
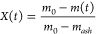
1

In order to quantify the reactivity of the samples, these conversion curves were fitted using the prominent Random Pore Model (RPM; eq 2) accounting for changes of the pore structure during char conversion.^43,53,54^
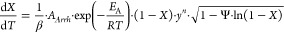
2

The change in conversion depends on the heating rate calculated with eq 3 as well as a reaction constant *k*, described by a pre-exponential factor *A*_*Arrh*_, an apparent activation energy *E*_*A*_, and the universal gas constant *R* according to Arrhenius.^55,56^
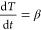
3

Conversion is modeled to be slower, the more char has already been consumed and to be faster, the higher the availability (mole fraction) *y* and the higher the apparent reaction order *n* of the reactive gas. Further, the RPM considers conversion-dependent pore evolution by including a structural parameter Ψ. The structural parameter accounts for an increasing char reactivity at the beginning of conversion but when the coalescence of pores happens with progressing conversion, the surface area decreases and, therefore, also reactivity.^10^

In order to simplify the fitting and reduce uncertainties due to a large amount of variables, the following values were taken as fixed parameters for the doped MH800 char samples obtained from previous fitting results of isothermal measurements in the same setup for the undoped reference MH800:^49^*A* = 9 × 10^8^ min^–1^; *n*_*O*_2__ = 1.14; *n*_*CO*_2__ = 0.51.

These values were taken for all the differently doped MH800 samples, assuming the doping to affect only the apparent activation energy and the structural parameter.

For the MH hydrochar samples, the kinetic parameters to be fixed were determined analogously to ref (49) as shown in the Supporting Information (SI) section S4. The modeling of TP curves was then performed using the falling branch of the conversion peak to limit the influence of overlapping devolatilization. A validation of this procedure by isothermal measurements is also shown in section S4.

Generally, fitting of the TP curves was achieved by least-squares fitting based on the interior-point algorithm in MATLAB R2020b with 1 × 10^6^ as the maximum number of iterations, complying to a tolerance of 1 × 10^–10^. The fit quality *R*^2^ relating experimental values (Exp.) to fitted values (Fit) was derived from eq 4 for all measurement points *z* of the experimental curve.
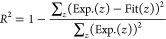
4

#### 2.3.2 Fitting of Loading Dependence

The loading-dependent behavior of the apparent activation energy as derived by fitting of the DTG curves with the RPM approach was also fitted to quantify the dependence. A Langmuir-type equation known to describe saturation behavior was used:
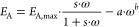
5

This equation expresses the maximum achievable influence *E*_A,max_ as being approached as a function of loading *w* with a certain strength of correlation *s*. For the hydrochar samples *M*-MH, a power law-type deactivation term with factor *a* and exponent *b* was additionally required to model the loading dependence. Again, least-squares fitting based on the interior-point algorithm was performed in MATLAB R2020b with 1 × 10^6^ as the maximum number of iterations, complying to a tolerance of 1 × 10^–10^. The fit quality for both parameters was obtained similarly to eq 4. Achieved fit qualities were in the range of 0.993–0.999 with the exception of Mg-MH in CO_2_ (0.751) and Ca-MH in O_2_ (0.888).

## 3 Results and Discussion

### 3.1 Reactivity Effects of Mineral Doping

**Figure 1:**
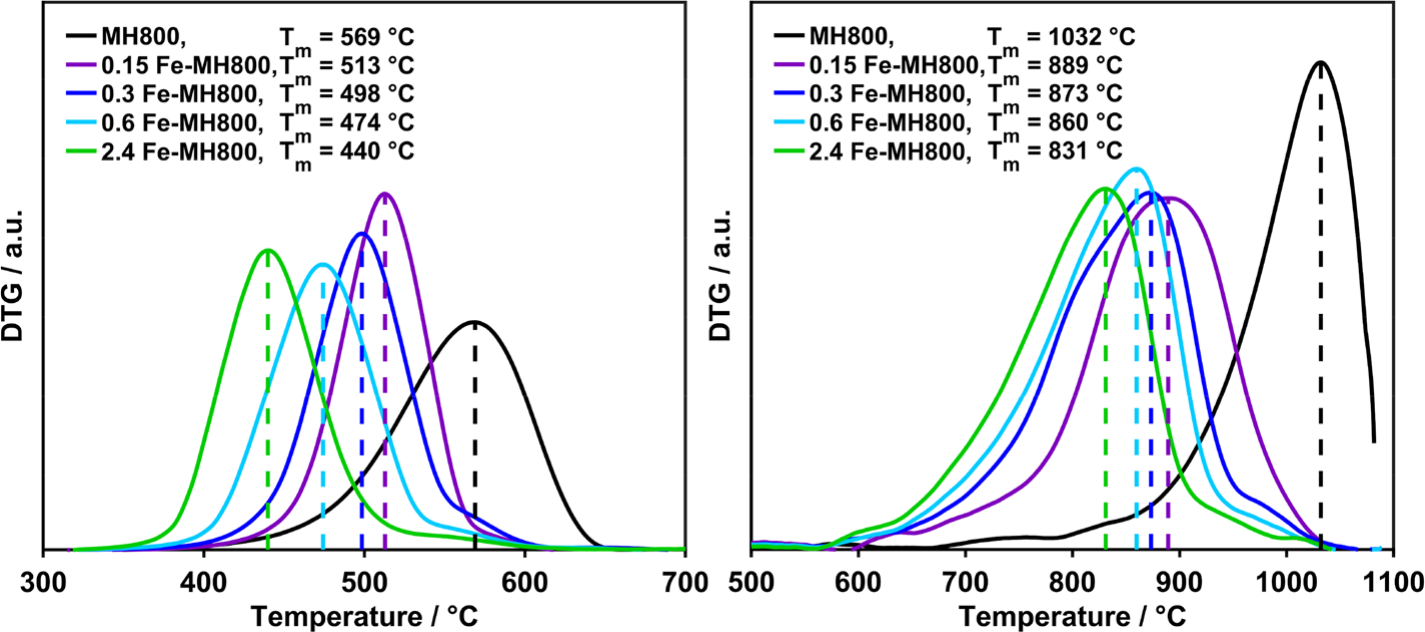
DTG curves of the TP measurements of Fe-MH800 in 20% O_2_/He (left) and 50% CO_2_/He (right).

The DTG curves of the TP experiments using the char sample MH800 in O_2_ are shown in Figure 1 (left) for Fe as well as in Figure S1 for the additional loading series. The subsequent discussions focus on the peak maximum as marked for the individual curves. For all loading series, doping resulted in a shift of the DTG peak maximum toward lower temperatures. This is in agreement with literature results showing a relative lowering of oxidation temperatures for natural biomass char samples in comparison to their washed analogues.^6^ While these literature studies only accounted for the overall shift due to contained minerals, specific and quantitative correlations are possible based on the selective doping in this work. To a certain point, the temperature shift was even more pronounced for increased loadings, indicating a loading dependence of the char oxidation reactivity within each metallic inorganic element series.

**Figure 2:**
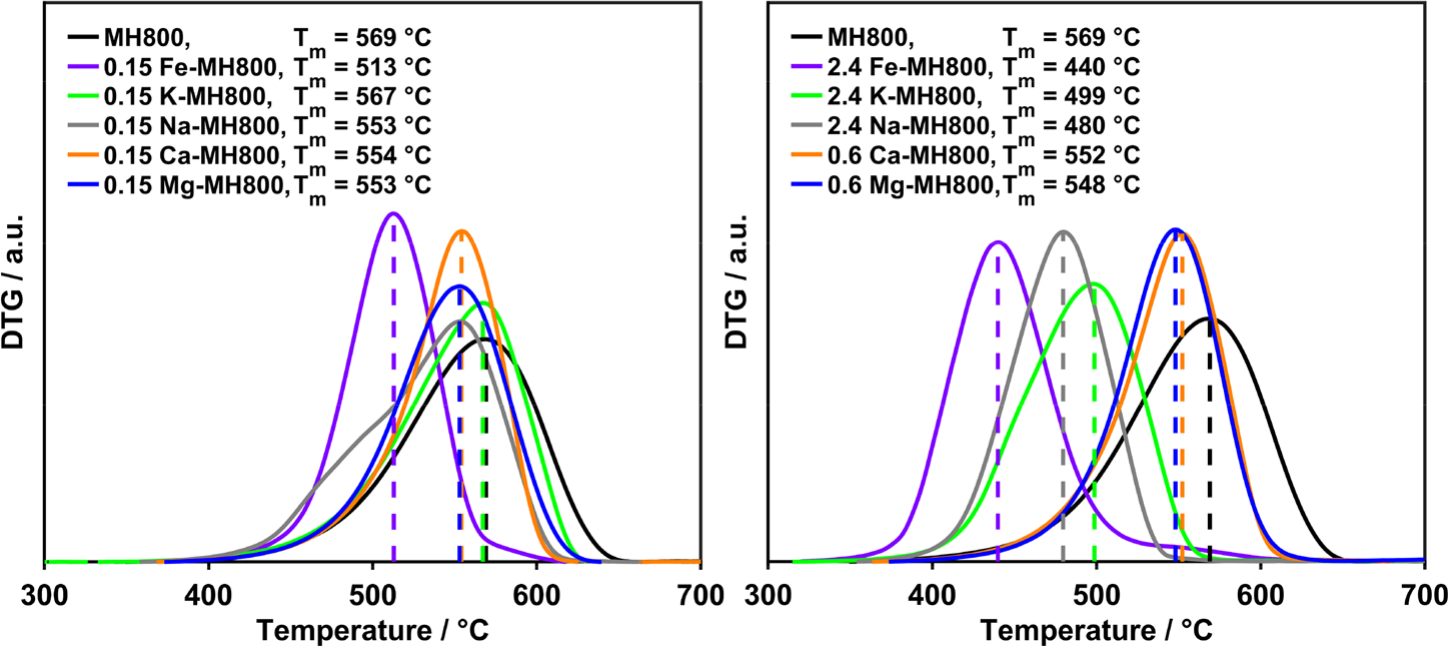
Comparison of metallic inorganic element reactivity in the oxidation of MH800 for different loadings (left: minimum loading, right: maximum loading).

However, as the increase was not continuous, a linear correlation was excluded, and further quantitative analysis is performed in section 3.2. Comparing the different dopants (Figure 2), the reactivity order was also found to be loading-dependent, indicating a differing strength of loading dependence for the different metallic inorganic elements.

At higher loadings, the highest char oxidation reactivity was found for Fe followed by the alkali metals, whereas the effect of the alkaline earth metals was the least pronounced. This comparable low reactivity of alkaline earth metals at higher loadings is due to the stagnation of the reactivity increase upon increasing the loading to more than 0.3 and 0.6 wt % for Ca and Mg, respectively. Although Fe clearly was the most active even at low loadings, the reactivity differences of alkali and alkaline earth metals are very small. For the 0.15 wt % loaded samples, oxidation of the samples doped with Na, Ca, or Mg proceeds in the same temperature range, whereas K is less reactive, exhibiting only a shift which is hardly significant.

**Figure 3:**
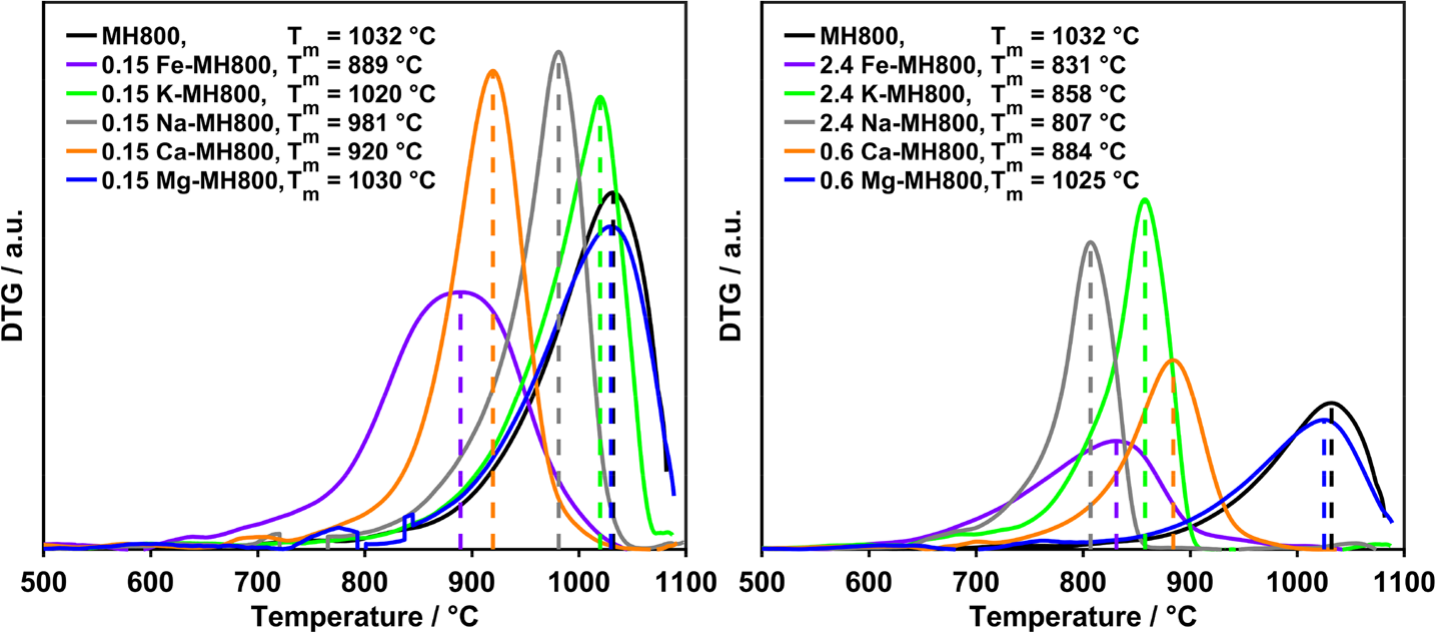
Comparison of metallic inorganic element reactivity in the gasification of MH800 for different loadings (left: minimum loading, right: maximum loading).

Upon gasification in CO_2_, the char reactivity increase due to doping as displayed in the obtained DTG curves (Figures 1 (right) and S1) was directly observed to a differing extent. As for oxidation, the temperature shift with metallic inorganic element loading corresponds to lower gasification temperatures when comparing natural chars with inherent mineral content to acid-washed chars.^57^ Within one metallic inorganic element series, the trend of an increased curve shift toward lower temperatures with an increased loading to a certain extent was similar to the oxidation curves, but the relative reactivity was different (Figure 3).

Remarkably, Ca was much more active in gasification than in oxidation, and the overall highest reactivity was obtained for Na instead of Fe. However, at the lowest loading of 0.15 wt %, Fe is still the most active metallic inorganic element followed by Ca, Na, K, and finally Mg. With increasing loading, the latter exhibits almost a stagnation in reactivity increase, whereas an intermediate loading dependence is found for Fe and Ca as well as an even strongly pronounced dependence for the alkali metals (Figure S1). In addition to differences of inherent biomass reactivity and counterions of dopants, the different reactivity changes for the various metallic inorganic elements may explain the existence of different reactivity orders for the investigated metallic inorganic elements in literature if only compared at a certain loading.^10,30,31,49^

**Figure 4:**
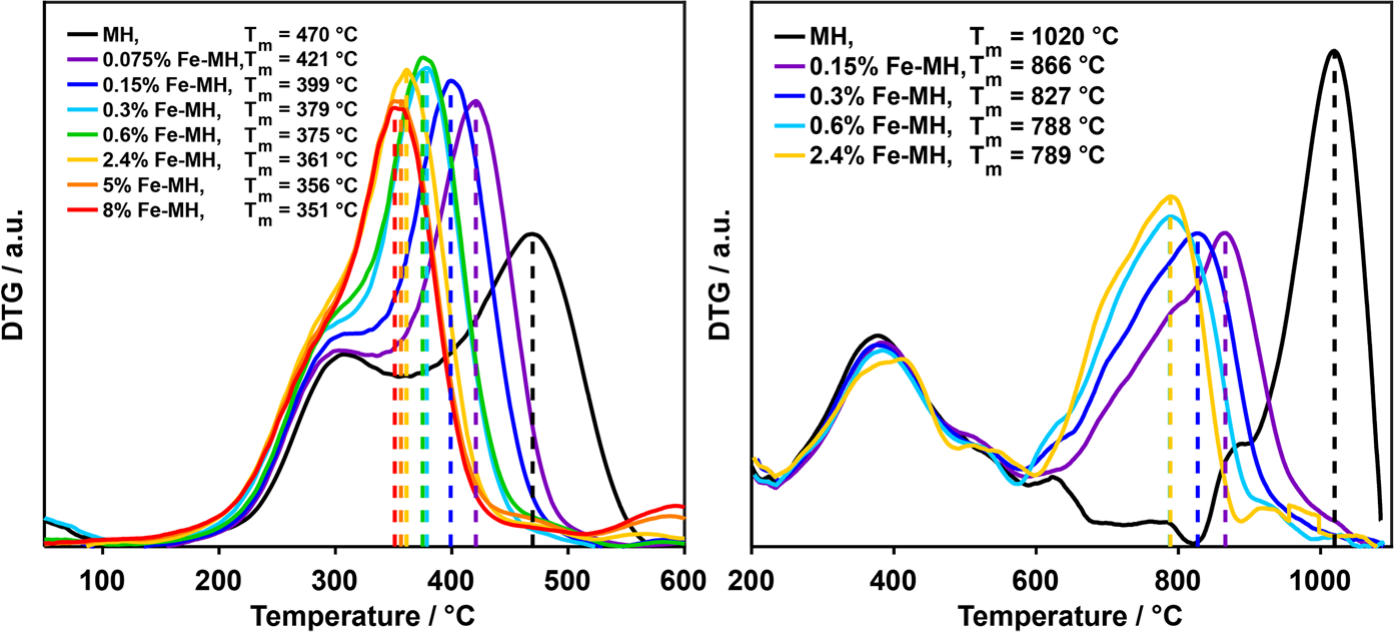
DTG curves of the TP measurements of Fe-MH in 20% O_2_/He (left) and 50% CO_2_/He (right).

The DTG curves of the experiments of doped hydrochar samples (MH) in 20% O_2_/He are shown in Figure 4 (left) for Fe and in S2 for the different other metallic inorganic element loading series. Independent of metallic inorganic element type or loading amount, the catalytic influence on the devolatilization temperature was negligible in all slow heating thermogravimetric measurements. In contrast, a comparison of the relative shift of the char conversion peak maximum temperature by doping revealed an increased char reactivity with increasing metallic inorganic element loading similar to the MH800 samples. Even small loadings of 0.15 to 0.3 wt % as found in natural biomass already showed a significant influence on char oxidation temperatures with temperature shifts of up to 90 °C, highlighting again the importance of including catalytic effects into models. The larger loading range revealed not only a saturation behavior as for the char but also differences in this loading dependence for the different investigated metal sulfates. For Fe, a higher loading increased the shift of the peak maximum temperature by steadily decreasing the temperature shifts even up to loadings of 8 wt %. In contrast, for the AAEM a maximum was reached between 2.4 and 5 wt % with further added minerals exhibiting either no (5% Na-MH, 10% Mg-MH) or even negative effects on the temperature shift (8% K-MH, 5% Ca-MH).

This generally observed saturation behavior is in agreement with literature reports.^14,15,17−21^ For example, increasing the doped amount of NaCl resulted in smaller and smaller effects on the yields of levoglucosan and glycolaldehyde during pyrolysis of cellulose.^20^ Guizani et al.^21^ reported “near-linear” correlations between beechwood char reactivity and metallic inorganic element molar concentrations up to 1 mol % during gasification. However, especially for Ca and K, the increase in metallic inorganic element loading did not increase the rate linearly at higher loading amounts. Possible explanations are the generation of multilayer deposits of the metal sulfates with limited contact to the carbon fuel or the blockage of the pore structure impeding the adsorption of reactive gas atmosphere on the inner carbon surfaces. Upon removal of water as the impregnation medium, dissolved species adsorb on the carbon surface. In this process, the dispersion and the distribution of the deposited particles on the carbon matrix depend on the strength of interaction and the amount of minerals relative to the available carbon surface. The ionic mineral species interact strongly with each other favoring the adsorption on already present mineral deposits especially for higher loadings compared with the adsorption directly on the carbon matrix. In this way, multilayer deposits are formed, the so-called agglomerates, instead of finely dispersed mineral particles distributed all over the surface. The loss of carbon material during thermal treatment may then lead to additional particle accumulation, resulting in agglomerates of further increased size.

Analogously, the DTG curves of selected experiments of doped MH performed in 50% CO_2_/He for the different metallic inorganic element loading series are shown in Figure 4 (right) for Fe and in S2 for the different other metallic inorganic element loading series. Again, independent of metallic inorganic element type or loading amount, the catalytic influence on the devolatilization temperature was negligible in all slow heating thermogravimetric measurements. However, due to the gasification reaction initiated at higher temperatures compared with the oxidation reaction, devolatilization peaks between 250 and 600 °C and char gasification peaks between 650 and 1100 °C were better separated. Clear differences between the catalytic effects on char conversion in oxidizing and gasifying atmospheres were visible. On the one hand, in 50% CO_2_/He, Fe already saturated between 0.6 and 2.4 wt % does not shift the peak temperature any further, while the AAEM (with Mg as an exception) also exhibited a saturation behavior but with steadily decreasing peak maximum temperatures up to a loading of 2.4 wt %. The peak shape was identified as another difference between Fe and the AAEM during the gasification measurements. While the AAEM all displayed sharp gasification peaks, the Fe signal showed a clear shoulder at lower temperatures, separating the gasification into two overlapping processes. Here, the multistep (partial) transformation of the Fe mineral phase already observed for the hydrochar in ref (5) and reported in literature for different carbon materials^58,59^ may cause the simultaneous or subsequent existence of different Fe phases with varied catalytic activities. Hence, multiple differently catalyzed gasification reactions may explain the broad gasification peaks of the Fe-doped samples at elevated temperatures compared with the oxidation measurements. Consistent with the char samples, the catalytic influence of Ca was strongly dependent on the applied atmosphere. In contrast to oxidation, in which the catalytic influence of Ca was comparably small, in gasification Ca demonstrated a very strong catalytic effect, especially at the lowest loading of 0.15 wt % resulting in a temperature shift of 139 °C. This is in agreement with literature reporting a high catalytic activity of Ca during biomass gasification.^34^ In general, the reactivity sequence of the metal sulfates in char gasification depended much more on the doping amount than during oxidation. At low loadings of 0.15 wt %, Fe and Ca caused the highest shift of the peak maximum temperature, while at high loadings of 2.4 wt % the alkali sulfates exhibited the strongest influence on the gasification temperature.

### 3.2 Loading Dependence of the Apparent Activation Energy

**Figure 5:**
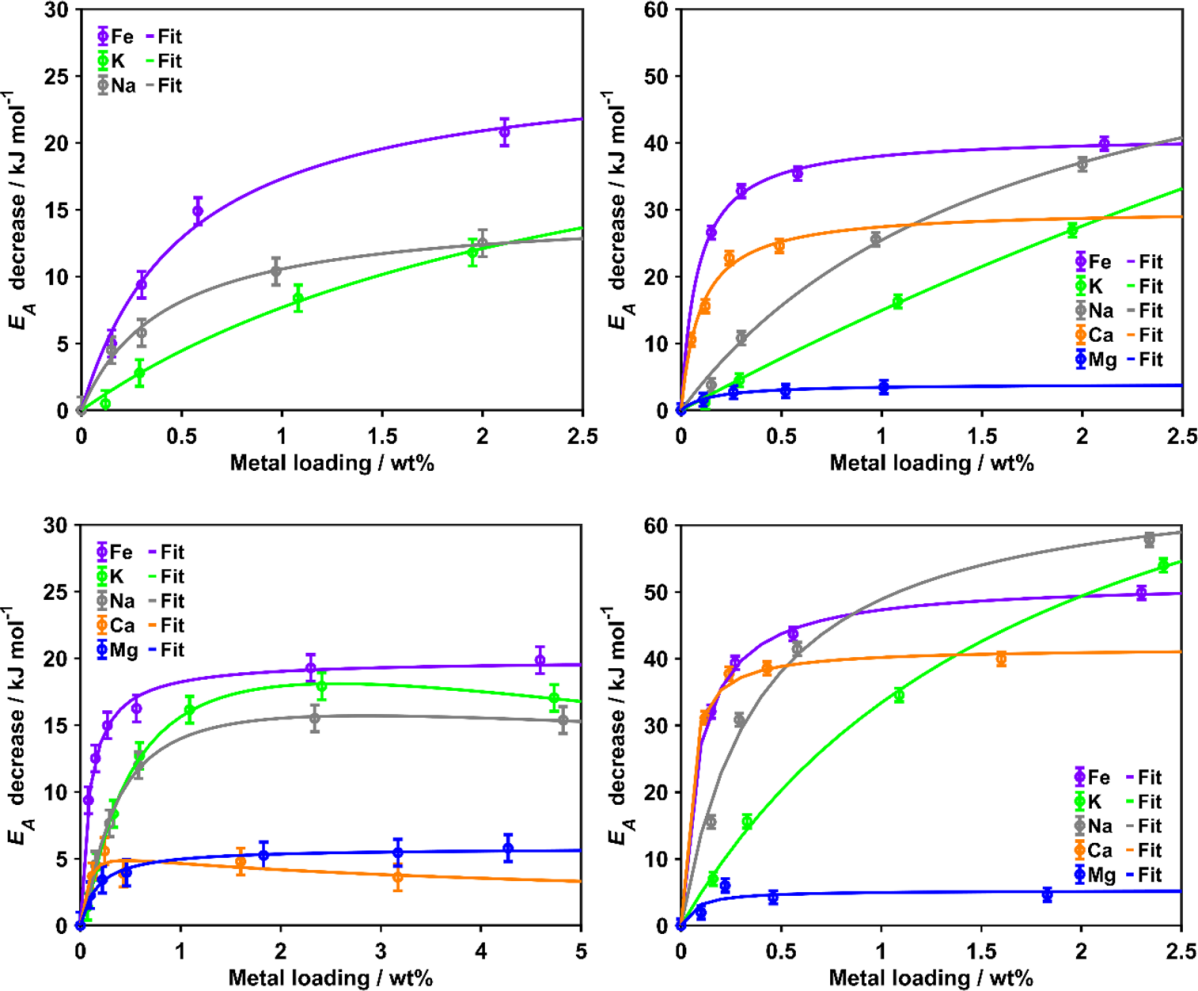
Loading dependence of the decrease in apparent activation energy *E*_A_ for the doped chars (top) and hydrochars (bottom) upon oxidation (left) and gasification (right).

In order to quantify the catalytic effect of the different samples in more detail and to simplify the material comparison, the apparent activation energy *E*_A_ decrease relative to the corresponding undoped sample as the decisive parameter is considered in the following. Consequently, the loading dependence was derived by correlating these values obtained from the DTG curve fitting with the actual loadings determined by AAS for the different metallic inorganic elements (Figure 5). Remarkably, for all investigated loadings of Mg and Ca on the char MH800, no resolvable effect on *E*_A_ was observed for the oxidation reaction. Besides these two series, a nonlinear loading dependence was found for the decrease in *E*_A_ in agreement with the trends observed for the overall reactivity directly displayed by the DTG curves. For all metallic inorganic element series showing a decrease of *E*_A_, changes were more pronounced at lower loadings, resulting in a nearly diminishing effect of further loading and, in case of the higher loadings on the hydrochar MH, even a reverse effect, representative of a deactivation by a too pronounced loading of minerals. The saturation effect of the *E*_A_ decrease accounted for by the Langmuir-type modeling occurred with differently pronounced strengths. The decrease was found to be about twice as strong for gasification than for oxidation independent of the fuel type. In addition to differences in the maximum achievable kinetic effect, fitting demonstrated a varied loading dependence for Fe and alkali metals in the two different atmospheres as well as for all metallic inorganic elements within one atmosphere. Consequently, the *E*_A_ decrease of the different metallic inorganic element loading series can be characterized by the maximum achievable decrease and the strength of loading dependence, both derived from the Langmuir-type fitting and summarized in Tables S5 and S6.

Focusing first on the detailed trends for the doped MH800 chars upon oxidation as well as gasification, the overall largest kinetic effect is expected for K doping. However, due to the low loading dependence, this only becomes decisive at higher loadings. At lower loadings, Fe shows the strongest effect despite its maximum achievable decrease of only intermediate strength, as it has a pronounced loading dependence, especially in gasification. Similar loading dependencies but lower maximum achievable decreases were found for Na in case of oxidation and Ca in case of gasification, therefore resulting in similar curve trends for these chars. Contrarily, the curve of Na-MH800 related to the gasification reactions proceeds rather similarly to the respective curve of K-MH800, as the loading dependence is almost equally low. Mg only exhibiting a small kinetic effect in char gasification is of negligible catalytic importance. Despite the intermediately pronounced loading dependence, its influence is limited by the low maximum achievable decrease of *E*_A_.

Several similarities to the previously described trends for doped chars can be found for the doped hydrochars. Upon oxidation, this comprises the most pronounced effects for the Fe-doped samples, followed by the alkali metals in a similar manner regarding both maximum effect and strength. This also holds for gasification, in which the two alkali metals are of a high maximum effect and low loading dependence so that their catalytic effect on char conversion becomes dominant at high loadings. In contrast, Fe and Ca dominate at low loadings typical for the metallic inorganic element content of natural biomass despite their lower maximum achievable effects as they have a higher loading dependence. Such a comparable high dependence is also found for Mg, but with a very low maximum effect. Focusing only on the maximum achievable effect on conversion of both fuels in both atmospheres, a reactivity sequence of K > Na > Fe > Ca ≫ Mg was found. Although the absolute effect is about two-three times higher upon gasification, the relative values are similar to oxidation.

Qualitative differences in the two materials are mainly related to the aforementioned absence of an *E*_A_ lowering for the oxidation of Ca- and Mg-MH800 and the deactivation in the hydrochar samples found for the alkali metals and Ca upon oxidation. Deactivation was found to be most prominent in the case of K, with higher loadings of K seeming to have limited the mobility of K species, for example, by the formation of agglomerates from large amounts of mineral particles during impregnation. Similarly, a strong inhibition as observed during oxidation of Ca-doped MH was also found for Ca-catalyzed coal gasification, in which a low loading of 1 wt % was found to be the optimum.^18^ This behavior indicates that an especially strong degree of contact between the carbon matrix and Ca is needed or that the activity of Ca atoms in multilayers or larger agglomerates formed during impregnation of higher loadings is significantly reduced. Potentially, deactivation with increasing loading as observed for oxidation of MH may also occur in the conversion of MH800 or during gasification of MH at higher loadings than investigated. For example, during gasification of alkali metal doped sawdust Kirtania et al.^14^ first observed a linear increase in reactivity with increasing loading before reaching a plateau followed by a decrease in reactivity at loadings higher than 3–4 wt % (metal/carbon molar ratio of 0.1). However, of most interest is the loading regime in which metallic inorganic elements are also abundant in natural biomass.

In this lower loading range, both the maximum effect and the strength of loading dependence are decisive and quantitative differences related to the different fuels as well as the different atmospheres are observed in addition. A very high agreement of parameters was found for the relative maximum achievable effects for MH upon comparing the two atmospheres and further when comparing the gasification of the two fuels except for K. Consequently, the influence of minerals on devolatilization mainly alters the oxidation behavior of the char, resulting in the observed differences between MH and MH800 when converted in diluted O_2_. The stronger effects for MH may be explained by the presence of minerals activating the carbon matrix during devolatilization.^60^ As the only exception, no difference in the char oxidation behavior was found for the Fe-doped samples. For this metallic inorganic element, the catalytic effect on char conversion is commonly assumed to be primarily based on RedOx cycles, whereas there are additional electronic effects discussed for AAEM. Following this distinction, the presence of minerals during the devolatilization of MH can be concluded to result in AAEM acting more strongly as electronic promoters.^37,61−63^ Differences in the catalytic mechanisms of Fe and AAEM may also explain the consistently high catalytic activity of the Fe-doped samples in this work. An activation of gaseous reagents by Fe minerals traversing RedOx cycles may be more favorable than electronic promotion by AAEM. Furthermore, studies of the thermally treated pure sulfate salts revealed the occurrence of a phase transition upon oxidation or gasification up to 800 °C only in the case of the Fe sulfate.^5^ Thus, Fe sulfate is the mineral most prone to oxidation, which is most likely to undergo phase transitions in a RedOx cycle as proposed to be mainly decisive for catalytic activity. In contrast, the sulfates of AAEM were found to be more stable, which may impede the conversion to catalytically active key species such as M_2_CO_3_, M(g), M_2_O, and MOH.^36^ Changes in the stability of mineral phases can be induced by close contact with the carbon matrix,^64^ and more detailed information on the chemical state of the minerals decisive for the reactivities obtained in this work would require *in situ* studies comprising bulk- and surface-sensitive characterization at different points in the sample history of both oxidized and gasified chars.

Furthermore, the similarity when converting both fuels in diluted CO_2_ indicates that the mineral effects initiated during devolatilization do not lead to an observable change in the gasification behavior. As gasification proceeds several hundred degrees above oxidation, these catalytic effects are superimposed by the thermal annealing of the carbon matrix.

The various trends observed for both hydrochar and char can be compared to exemplary studies in literature. Reported loading dependencies were based on different biomasses, minerals, and loading ranges. In the case of oxy-fuel conversion, the catalytic effect of different K salts was investigated.^11^ For KCl and K_2_CO_3_ saturation occurred at about 2 and 3 wt %, respectively, while K_2_SO_4_ showed a linear increase up to the maximum loading of 4 wt %. This fits well to the saturation curve obtained here, which was shown to be in the initial range and changes almost linearly with conversion. Similarly, for gasification the reactivity evolution of biomasses impregnated with K_2_CO_3_ was found to proceed linearly for loadings of up to 2 wt %.^13,65^ A series of more strongly varied alkali metal loadings indicated the saturation with K to occur above 10 wt %, whereas with Na saturation is already expected above 6 wt %.^14^ These different saturation ranges can be explained by the Langmuir-type fitting parameters obtained in this work, as the lower loading dependence in case of K leads to saturation expected at much higher loadings than for Na. In addition to the alkali metals, the loading dependence of Ca and Fe in gasification was exemplarily studied in literature.^13,32^ In the latter case, saturation occurred when doping about 2 wt % as chloride, which correlates well with the curves in Figure 5 top and bottom right. Conversely, for CaCO_3_ loadings of up to 0.5 wt %, literature still reports a linear behavior, while beginning saturation is already observed here.^65^ Regarding a quantification of the activation energy decrease, the derived Δ*E*_A,max_ for MH800 and MH of K (178 and 94 kJ mol^–1^) and Na gasification (both 68 kJ mol^–1^) are very similar to values of 104 and 53 kJ mol^–1^ (refs (10) and (31), respectively) obtained by applying the RPM to the gasification of southern pine (K) and pistachio nutshells (Na), respectively.

Overall, the systematic evaluation of the loading dependence derived for the activation energies for both solid fuels in this work is in good agreement with literature studies despite the differences in used materials. This points toward a transferability of established relationships for the different types of metallic inorganic elements with regard to their relative as well as absolute behavior upon varied loadings in both oxidation and gasification. In conclusion, the investigated fuels seem to be suitable model biomasses to study the catalytic influence of minerals.

### 3.3 Structural Parameter

In addition to *E*_A_, the structural parameter Ψ was also determined for the different metallic inorganic element samples. A fluctuating behavior within 1 order of magnitude was found in the loading series of MH and MH800 in both atmospheres. Averaged values are summarized in Table 1. Ψ describing the porosity of the solid fuel is close to zero for samples with high porosity in which conversion proceeds overall in the sample volume including smaller pores^14,65^ and increases if conversion only takes place in larger pores or if pores are blocked by minerals. However, for the investigated loading series only at the extremely high loadings of 8 or 10 wt % measured in oxidation Ψ increased strongly, highlighting that severe blockage of pores did not occur in the majority of the investigated doping range. In contrast, the overall increase observed when comparing the doped samples to the undoped samples, as well as gasification to oxidation, and MH800 and MH can be related to the following effects. Upon impregnation, the minerals may agglomerate in the pores, thereby decreasing the char reactivity by hindering the diffusion of the gaseous reactants to the carbon active sites.^8,66^ This is more severe in case of oxidation, as here the reaction is assumed to proceed also in micropores, whereas gasification mainly occurs in larger mesopores.^21,67^ Combining these effects with the lower relative presence of mesopores in MH800 compared with MH, the loading of a similar amount of minerals affects the char sample more strongly. A decrease in the structural parameter as observed for Fe-doped samples during gasification can be explained by the possible generation of new active sites at the catalyst/carbon interface.^68^ However, the change of the structural parameter in CO_2_ was much smaller than during oxidation indicating that independent of the doping the preference of CO_2_ to react in large pores still dominated the reaction pathway. Still, as the accumulation of minerals in pores mainly affected the structural parameter of oxidation, it can be assumed that the majority of the minerals was deposited in the smaller pores of the chars during the impregnation process.

**Table 1:** Averaged Structural Parameters Ψ Derived from the DTG Curve Fitting by RPM for the Loading Series between 0.15 and 5 wt % in Both Atmospheres and for Both Fuels

	MH		MH800	
metallic inorganic element	oxidation	gasification	oxidation	gasification
	7 × 10^–9^	1	0.05	1
Fe	2 × 10^–7^	0.01	3	1 × 10^–5^
K	2 × 10^–8^	5	0.6	11
Na	5 × 10^–7^	2	1	10
Mg	1 × 10^–4^	1	3	0.7
Ca	0.3	0.3	3	6

Overall, the systematic investigation of loading series of hydrochar and char upon oxidation and gasification resulted in a comprehensive quantitative assessment of the catalytic effects. The obtained results enable the implementation of mineral effects into common combustion models by providing a broad set of parameters. For an improved description of combustion kinetics, the activation energies of char conversion in models such as the CCK/G and the CRECK-S-B may be adapted based on combining the actual amount of individual metallic inorganic elements in the investigated fuel with the corresponding loading-dependent effect derived in this work. Similarly, an adaption of physical model extensions is possible by utilizing the derived structural tendencies.

## 4 Conclusions

The effect of systematic mineral doping on char reactivity with and without mineral-influenced devolatilization was investigated to adapt combustion models with the catalytic effects of minerals. Qualitative reactivity series of sulfate-doped samples were derived from temperature-programmed measurements in diluted O_2_ and CO_2_ shifted char conversion peaks in the DTG curves to lower temperatures. In order to quantify the catalytic effects, the DTG curves were fitted using the random pore model (RPM) to derive kinetic and structural parameters. Obtained activation energies did not show a general order of reactivity, as the catalytic effects of the metallic inorganic elements differed with loading. However, Fe sulfate was always among the highest active minerals, and alkali metal sulfates were typically more reactive than alkaline earth metal sulfates. The only exception was the high activity of very small Ca sulfate loadings during gasification. The derived loading dependence of the apparent activation energies showed a clear saturation behavior which was successfully described by a Langmuir-type equation extended by a deactivation term for K-, Na-, and Ca-containing minerals during oxidation. The fit parameters revealed the maximum effect on the apparent activation energy as well as the strength of loading dependence and enabled the prediction of the catalytic effect for any loading of these metallic inorganic elements during oxidation and gasification of both the hydrochar MH and the char MH800 up to loadings of 5 and 2.4 wt %, respectively. Following the quantification of these parameters, the fuel comparison revealed a similarity in the relative effects of the MH samples in the different atmospheres as well as when comparing the gasification of both fuels. Therefore, the presence of minerals during devolatilization mainly increases the oxidation reactivity with AAEM possibly acting reinforced electronically, while for gasification this effect was superimposed by thermal annealing. Similarly, changes in the structural parameter due to the mineral doping also mainly affected the oxidation taking place in the smaller pores, whereas only a metallic inorganic element loading higher than 8 wt % seems to have caused significant mass transport limitation by pore blockage.

## Supporting Information Available

Supplementary sample characterization, thermogravimetric measurements of the AAEM loading series, development and validation of fitting the hydrochar measurements, and detailed parameters of the Langmuir-type modeling of the loading dependence (PDF)
