# Supplementary material for: The Catalytic Effect of Iron and Alkali and Alkaline Earth Metal Sulfates Loading Series on the Conversion of Cellulose-Derived Hydrochars and Chars
Source: ACS Omega. 2023 Mar 10;8(11):10629–39. doi: 10.1021/acsomega.3c00887 (PMC10035008; doi:10.1021/acsomega.3c00887)
Supplement: Supplementary file 1 — ao3c00887_si_001.pdf [file ao3c00887_si_001.pdf]

Supplementary Information

The Catalytic Effect of Iron, Alkali and Alkaline Earth Metal Sulfates Loading Series on the Conversion of Cellulose-Derived Hydrochars and Chars

Till Eckhard‡, Christin Pflieger‡, Jannik Böttger, Pascal Telaar, Francesca Cerciello*, Martin Muhler*

‡These authors contributed equally.

* francesca.cerciello@ruhr-uni-bochum.de; muhler@techem.rub.de

Laboratory of Industrial Chemistry, Ruhr University Bochum, 44780 Bochum, Germany

S1 Sample Characterization

Table S1. Proximate und ultimate analysis of the hydrochar MH and the char MH800.

Sample	C / wt%	H / wt%	O* / wt%	$Volatiles_{db}$ / wt%	Fixed $Carbon_{db}$/wt%	Ash_{db} / wt%
MH	66	4.5	29.5	53	47	0
MH800	94.9	4.3	0.8	<1	>99	0

**Calculated as O = 100 – C – H. db dry basis.*

Table S2. Metallic inorganic element content of the sulfate doped MH800 samples determined by AAS (wt%).

Metallic inorganic element	Nominal metallic inorganic element weight fraction after impregnation in wt%					
	0.075	0.15	0.30	0.60	1.20	2.40
Fe	-	0.15	0.30	0.58	-	2.11
K	-	0.12	0.29	-	1.08	1.95
Na	-	0.15	0.30	-	0.97	2.00
Mg	-	0.11	0.26	0.52	1.01	-
Ca	0.05	0.12	0.24	0.49	-	-

Table S3. Metallic inorganic element content of the sulfate doped MH samples determined by AAS (wt%).

Metal	Nominal metallic inorganic element weight fraction after impregnation in wt%								
	0.075	0.15	0.30	0.60	1.20	2.40	5	8	10
Fe	0.08	0.15	0.27	0.56	-	2.30	4.59	7.24	-
K	0.07	0.16	0.33	0.59	1.09	2.41	4.73	7.77	-
Na	-	0.15	0.29	0.58	-	2.34	4.82	-	-
Mg	-	0.10	0.22	0.46	-	1.83	3.73	-	4.27
Ca	-	0.12	0.24	0.43	-	1.60	3.17	-	5.41

For doping with the sulfates of Fe and alkali metals, the obtained metallic inorganic element loadings were in line with the desired values, especially for loadings below 1 wt%. In contrast, the doping with the sulfates of Mg and Ca resulted in significantly smaller metallic inorganic element loadings than planned. A possible explanation for the Ca loading being lower than expected (impregnated in the form of $CaSO_4 \cdot 2\ H_2O$), is the lower solubility compared with the other metal sulfates. This low solubility forced the use of a bigger round bottle flask during the impregnation procedure and, therefore, a higher glass surface area was present increasing the amount of metallic inorganic element lost to competing adsorption sites. In contrast, $MgSO_4$ is highly soluble but only around 70% of the desired values were found in the hydrochar. In

general, the higher the target value was set, the more the determined metallic inorganic element content deviated indicating saturated surface areas/pores of the model fuel. Overall, the achieved loadings increased almost linearly with the targeted loading enabling quantitative comparisons within one loading series as well as between the different metallic inorganic element.

S2 Supplementary Thermogravimetric Measurements of the Loading Series

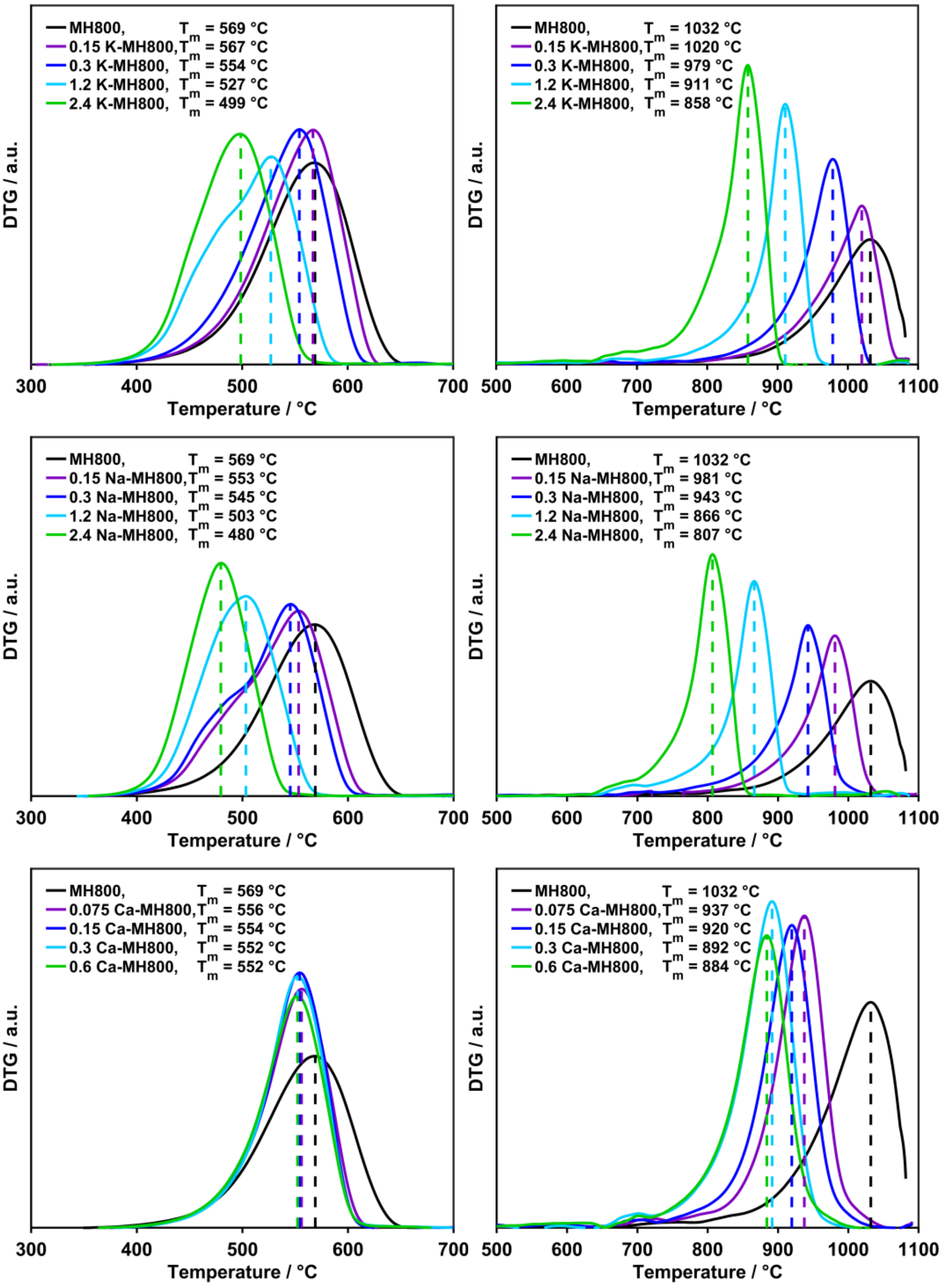

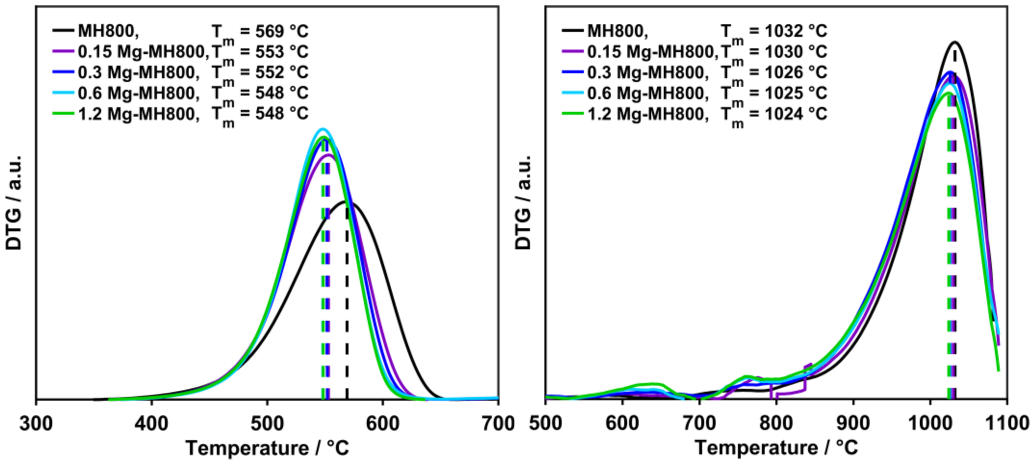

Figure S1. DTG profiles of MH800 doped with increasing loadings of individual metal sulfates measured in 20% O_2/He or 50% CO_2/He.

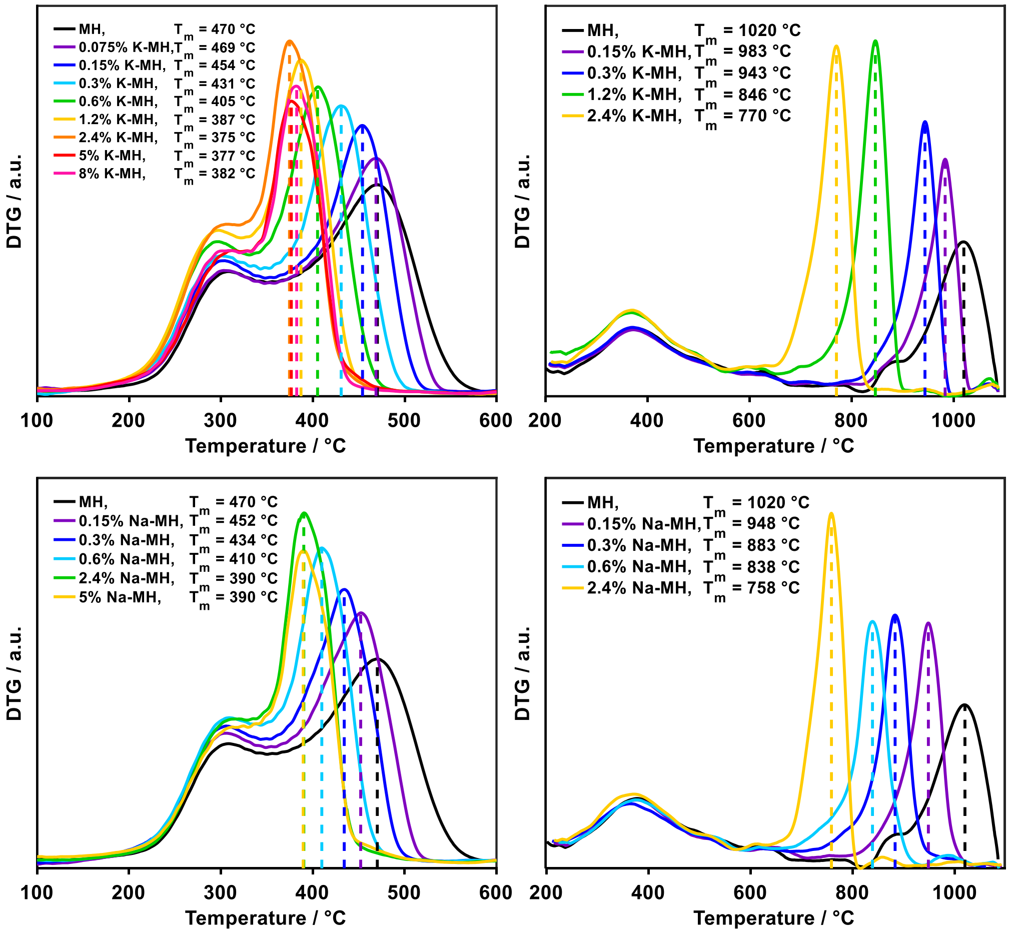

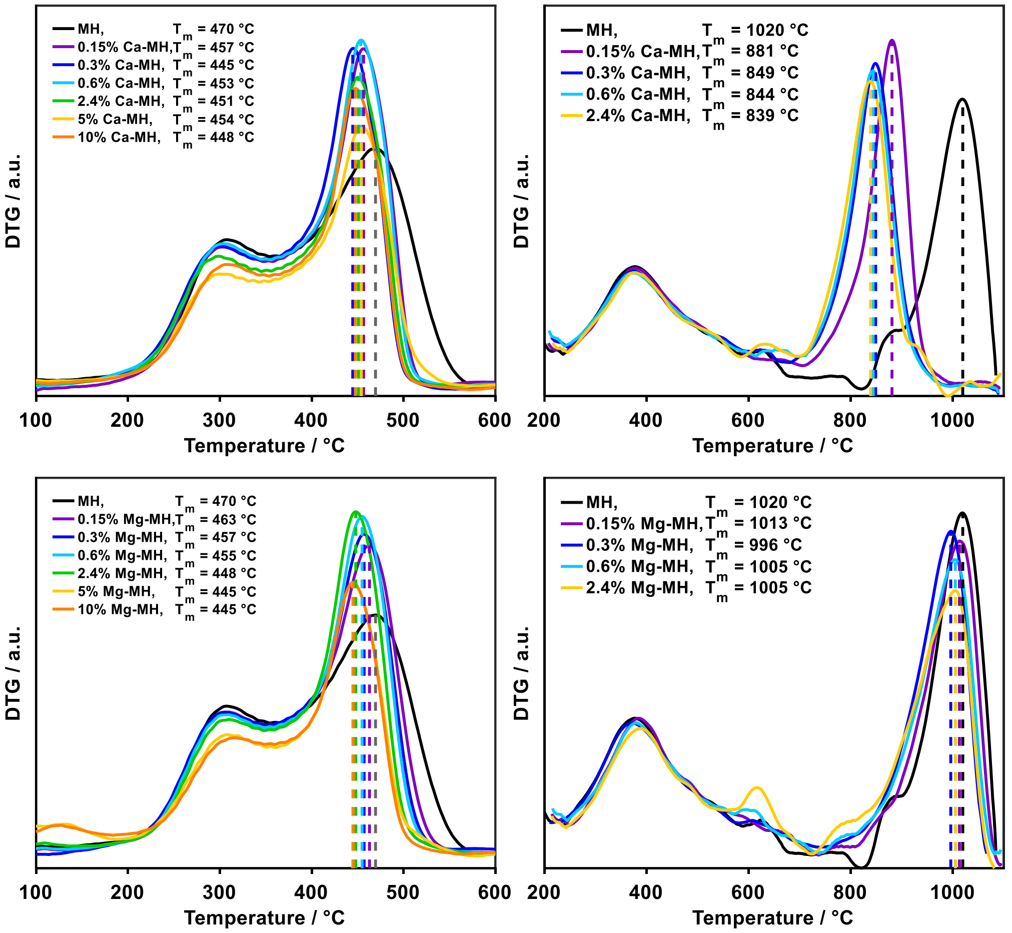

Figure S2. DTG profiles of MH doped with increasing loadings of individual metal sulfates measured in 20% O_2/He 50% CO_2/He.

S3 Validation of the MH800 fitting

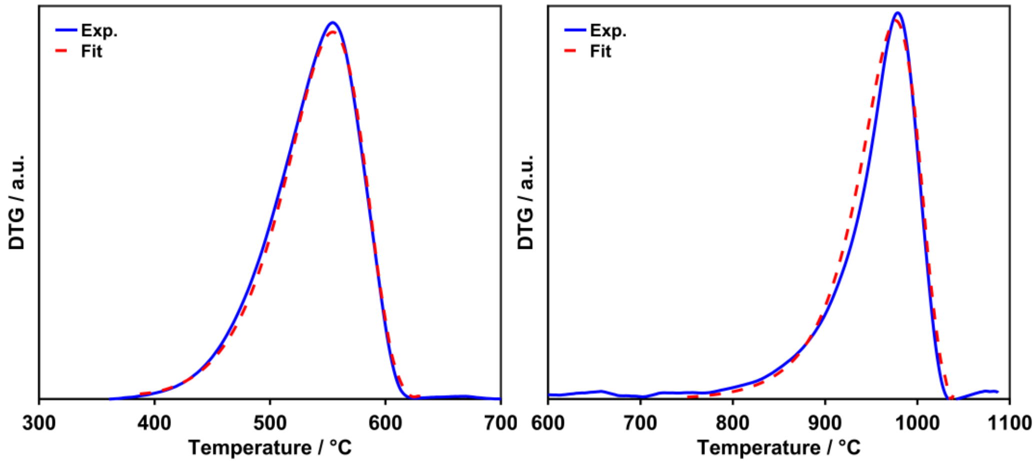

Figure S3. Comparison of experimental (Exp.) and fitted curve (Fit) for modelling the conversion of MH800 in diluted O_2 (left) and diluted CO_2 (right).

S4 Development and Validation of the MH fitting

First, isothermal mole fraction variations of O_2 or CO_2 in He for the undoped MH model fuel were performed at constant temperatures to derive the n_{O_2} and n_{CO_2} for oxidation and gasification, respectively. Fixing the derived apparent reaction orders, isothermal temperature variations at 20% O_2/He and 50% CO_2/He were used to derive kinetic parameters such as A_{Arrh} and E_A. Sets of three isothermal measurements obtained in both atmospheres applying varied mole fractions of reactive gas and temperatures were fitted using the RPM (equation (2)). Figure S4 shows the isothermal measurements performed for oxidation of the undoped MH fuel.

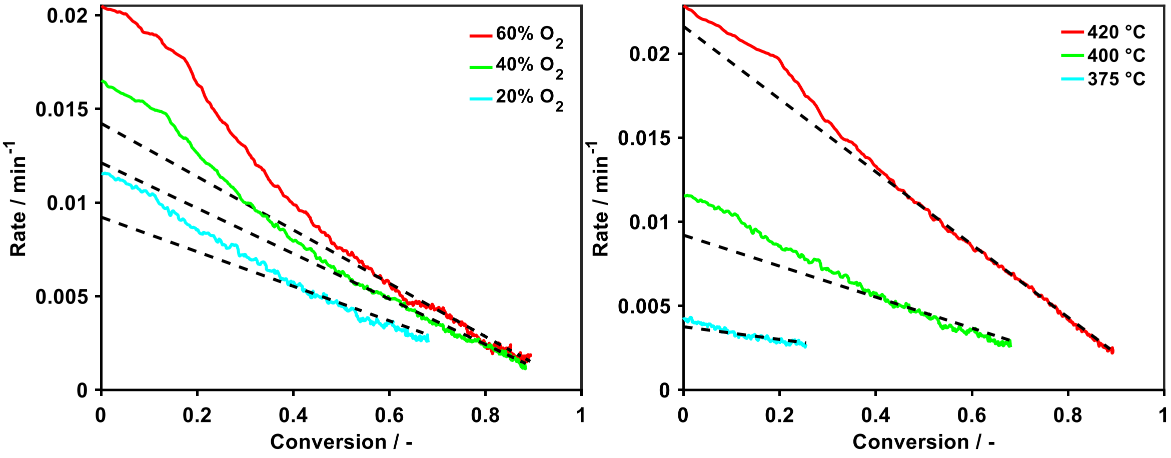

Figure S4. RPM fitted isothermal measurements of MH with an O_2 mole fraction variation at 400 °C (left) and a temperature variation in 20% O_2/He (right). RPM fit as dashed lines.

Clearly, the degree of char conversion at one temperature strongly depended on the applied concentration of the reactive gas. However, the full agreement between experimental data and fit was impaired by the simultaneous devolatilization during the first half of conversion. Nevertheless, in accordance with the 53 wt% of volatiles in the sample, the agreement of fit and experiment was much better for conversion degrees above 50 %. Hence, this range of the measurements was used for the quantification of the apparent reaction order for oxidation. The calculated n_{O_2} of 0.39 is slightly smaller compared with values reported for different carbon materials in literature between 0.5 and 1.0 and also smaller than the value reported for MH800 (n_{O_2} = 1.14) [1,2]. Here, the larger saturation of the carbon matrix with abundant oxygen

functional groups in the hydrochar may explain the difference in the apparent reaction orders between the hydrochar and the char. Fixing n_{O_2}, isothermal temperature variations at a constant molar ratio of O_2 were used to derive the pre-exponential factor A_{Arrh} and the apparent activation energy E_A, as well as the structural parameter Ψ.

The isothermal measurements in CO_2 atmosphere were analyzed applying the same procedure as shown in Figure S5.

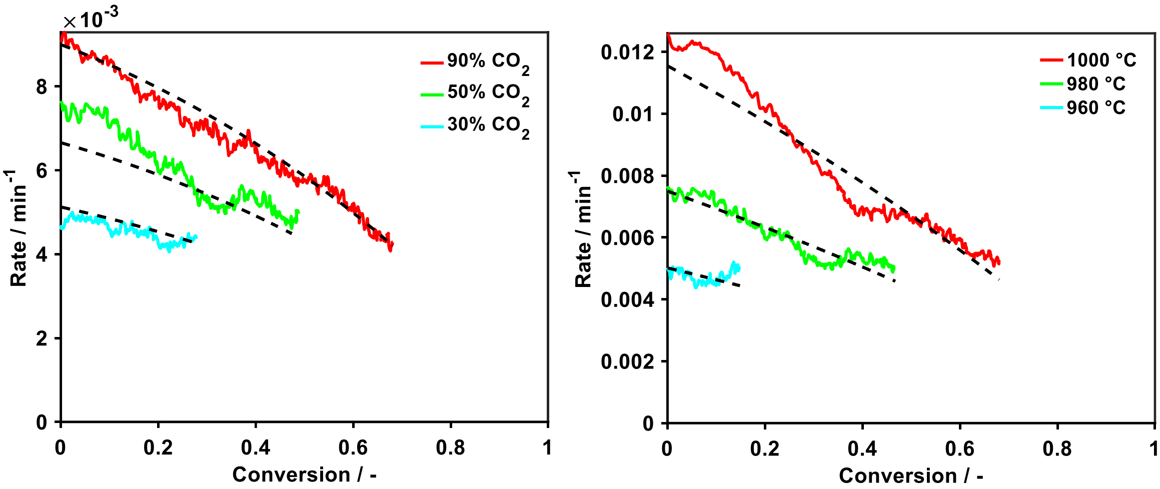

Figure S5. RPM fitted isothermal measurements of MH with a CO_2 mole fraction variation at 980 °C (left) and a temperature variation in 50% CO_2/He (right). RPM fit as dashed lines.

Here, the temperature separation of devolatilization and gasification observed in the DTG profiles resulted in a better agreement of fit and experimental values over the whole range of conversion. The resulting calculated kinetic and structural parameter as well as the apparent reaction orders for both atmospheres are summarized in Table S4.

Table S4. RPM fitting results of isothermal measurements of MH and 1.2% K-MH in oxidation and gasification atmospheres in comparison with the kinetic parameters derived from RPM fitting of the DTG curves.

Parameter	MH		1.2% K-MH	
	O_2	CO_2	O_2	CO_2
n	0.39	0.31	*2	*2
A_{Arrh} / min^{-1}	$4·10^8$	$4·10^9$	*2	*2
E_A / $kJ\ mol^{-1}$	132	278	120	236
Ψ	$2·10^{-8}$	0.5	$8·10^{-7}$	3
E_A (DTG)*1 / $kJ\ mol^{-1}$	132	278	116	242
Ψ (DTG)*1	$7·10^{-9}$	1	$2·10^{-8}$	5

*1 n and A_{Arrh} fixed from the isothermal measurements.

*2 Values fixed from undoped MH.

In accordance with literature [1], n_{CO_2} is smaller than n_{O_2} and only marginally smaller than the typically reported values for n_{CO_2} between 0.4 and 0.7 for various biomass chars. While the E_{A,O_2} of MH for oxidation amounting to 132 kJ mol^{-1} is 14 kJ mol^{-1} smaller than the derived E_{A,O_2} of the MH800 char, thereby describing a more reactive hydrochar compared with the char, the E_{A,CO_2} of MH for gasification is 39 kJ mol^{-1} larger than the corresponding E_{A,CO_2} of MH800. Nevertheless, the hydrochar is still also more reactive during gasification compared with the char, as the A_{Arrh,CO_2} of the hydrochar is two orders of magnitude larger than that of the char. Considering that CO_2 gasification takes mainly place in macro- und mesopores [3,4], the high contribution (almost 50%) of these large pores to the surface area of the hydrochar may explain the difference in A_{Arrh,CO_2} for both fuels. In comparison, in MH800 macro- und mesopores only contribute by around 7% to the overall surface area.

Concerning the structural parameter describing the porosity of the fuel, a higher Ψ is expected for gasification. A Ψ value close to zero describes a sample with high porosity in which conversion proceeds overall in the sample volume including smaller pores [5,6]. In contrast, if conversion only takes place in larger pores or if pores are blocked by minerals, an increase

of Ψ is expected, explaining the seven orders of magnitude difference between Ψ_{O_2} and Ψ_{CO_2} observed for the undoped MH fuel.

In order to limit the influence of overlapping devolatilization and char conversion in the isothermal measurements, especially during oxidation experiments, the RPM fitting of the falling branch of the DTG curves was tested. Another advantage of this method was the use of only one temperature-programmed measurement instead of the need to measure three isothermal measurements, saving a lot of time considering the number of doped samples.

Fixing n and A_{Arrh} derived from the isothermal measurements, the fitting results for the DTG curves of undoped MH in 20% O_2/He and 50% CO_2/He are summarized for comparison with the results of the isothermal measurements in Table S4. A good agreement of fit and experimental data was achieved as shown in Figure S6, deriving almost identical values for E_A and Ψ in both atmospheres compared with the isothermal measurements.

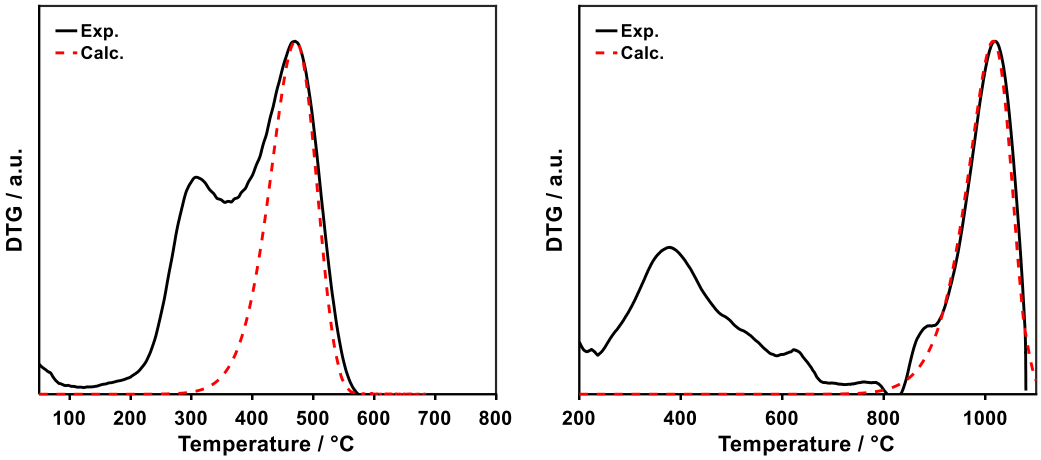

Figure S6. RPM fitted DTG curves of MH during oxidation in 20% O_2/He (left) and gasification in 50% CO_2/He using the fixed n and A_{Arrh} values derived from the respective isothermal measurements. RPM fit as dashed lines.

After validation of the DTG curve fitting by RPM also in the case of overlapping devolatilization providing similar kinetic and structural parameters as the isothermal measurements, 1.2% K-MH as an example for a doped sample with relatively high loading and significant catalytic effect was chosen to repeat the validation. Assuming that the doping with minerals only affects the apparent activation energy and the structural parameter, n and A_{Arrh} derived from the isothermal measurements of the undoped hydrochar were fixed. The analogous figures to Figure S5 and Figure S6 for 1.2% K-MH are shown in Figure S7 and Figure S8. For a better comparison, the calculated parameters derived from isothermal measurements as well as from DTG fitting are also summarized in Table S4.

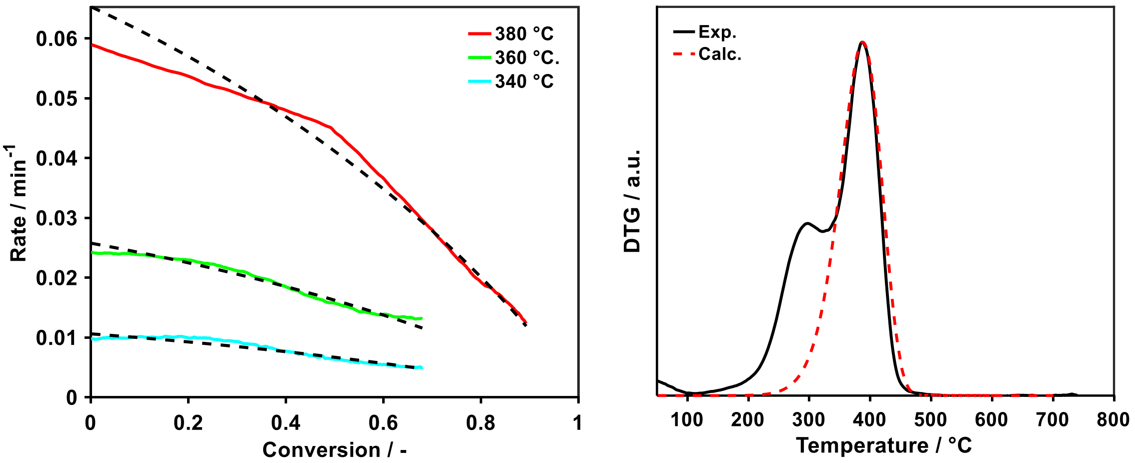

Figure S7. RPM fitted isothermal measurements (left) and DTG curve (right) of 1.2% K-MH in 20% O_2/He used to derive E_A and Ψ with fixed n and A_{Arrh} values of the undoped MH. RPM fit as dashed lines.

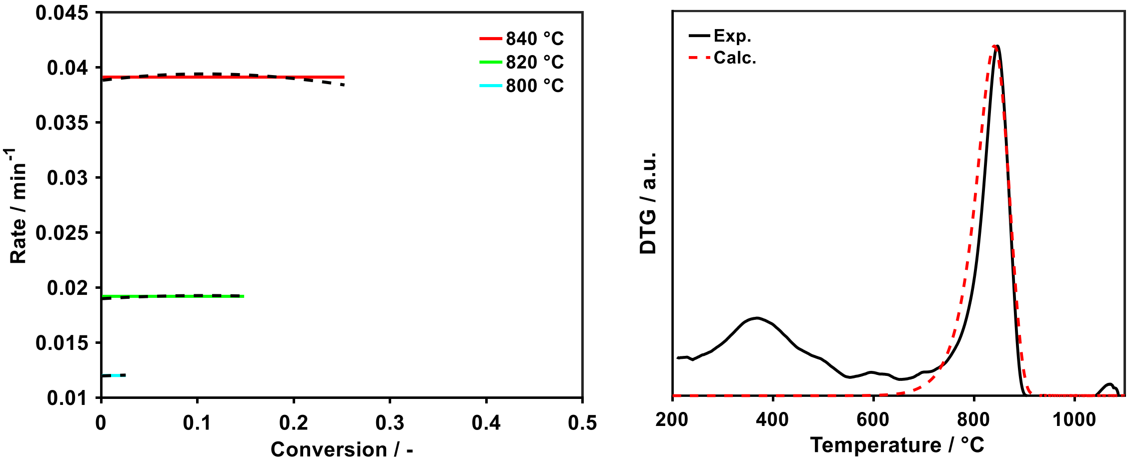

Figure S8. RPM fitted isothermal measurements (left) and DTG curve (right) of 1.2% K-MH in 50% CO_2/He used to derive E_A and Ψ with fixed n and A_{Arrh} values of the undoped MH. RPM fit as dashed lines.

While not as perfect a match as for the undoped sample, both fitting approaches resulted in similar E_A and Ψ for 1.2% K-MH in oxidation and gasification, respectively. Hence, the DTG fitting approach by RPM was successfully validated also for doped samples. Referring to the DTG fitting results in the following, the apparent activation energy decreased due to the catalytic effect of the 1.2 wt% K, resulting in a decrease by 16 kJ mol^{-1} (12%) during oxidation and a decrease by 36 kJ mol^{-1} (13%) during CO_2 gasification. As predicted earlier, the structural parameter of the doped sample slightly increased in comparison with the undoped sample

indicating that mineral deposits may inhibit mass transport. Nevertheless, it can be assumed that the reactions still take place in the whole volume of the sample, as the value is still close to zero.

S5 Parameters of the Loading Dependence

Table S5. Langmuir-type parameters of the maximum effect $\Delta E_{A,max}$ in kJ mol^{-1} (and its corresponding percentage compared with the undoped MH800 in brackets) and the strength of loading dependence s in wt%$^{-1}$ derived for the loading dependence of the activation energy difference ΔE_A during oxidation and gasification.

Metallic inorganic element	Oxidation		Gasification	
	$\Delta E_{A,max}$	s	$\Delta E_{A,max}$	s
Fe	26 (18%)	1.9	41 (17%)	12.3
K	28 (19%)	0.4	178 (74%)	0.1
Na	15 (10%)	2.4	68 (28%)	0.6
Mg	-	-	4 (2%)	6.7
Ca	-	-	30 (13%)	10.4

Table S6. Langmuir-type parameters of the maximum effect $\Delta E_{A,max}$ in kJ mol^{-1} (and its corresponding percentage compared with the undoped MH in brackets) and the strength of loading dependence s in wt%$^{-1}$ as well as the strength of loading-dependent deactivation a in wt%$^{-1}$ and the power b derived for the loading dependence of the activation energy difference ΔE_A during oxidation and gasification.

Metallic inorganic element	Oxidation				Gasification			
	$\Delta E_{A,max}$	s	a	b	$\Delta E_{A,max}$	s	a	b
Fe	20 (15%)	11.2	0	-	51 (18%)	11.2	0	-
K	38 (29%)	1.8	9	0.4	94 (34%)	0.6	0	-
Na	29 (22%)	2.9	7	0.3	68 (24%)	2.5	0	-
Mg	6 (5%)	6.3	0	-	5 (2%)	15.0	0	-
Ca	13 (10%)	25.0	7	0.1	41 (15%)	28.0	0	-

References

(1) Pflieger, C.; Lotz, K.; Hilse, N.; Berger, C. M.; Schiemann, M.; Debiagi, P.; Hasse, C.; Scherer, V.; Muhler, M. Catalytic influence of mineral compounds on the reactivity of cellulose-derived char in O_2-, CO_2-, and H_2O-containing atmospheres. *Fuel* 2021, *287* (1700–1729), 119584. DOI: 10.1016/j.fuel.2020.119584.

(2) Di Blasi, C. Combustion and gasification rates of lignocellulosic chars. *PECS* 2009, *35* (2), 121–140. DOI: 10.1016/j.pecs.2008.08.001.

(3) Butterman, H. C.; Castaldi, M. J. Experimental Investigation of Lignin Decomposition and Char Structure During CO_2 and H_2O/N_2 Gasification. *Waste Biomass Valor* 2012, *3* (1), 49–60. DOI: 10.1007/s12649-011-9086-2.

(4) Watanabe, H.; Okazaki, K. Effect of minerals on surface morphologies and competitive reactions during char gasification in mixtures of O_2 and CO_2. *Proc. Combust. Inst.* 2015, *35* (2), 2363–2371. DOI: 10.1016/j.proci.2014.06.119.

(5) Kirtania, K.; Axelsson, J.; Matsakas, L.; Christakopoulos, P.; Umeki, K.; Furusjö, E. Kinetic study of catalytic gasification of wood char impregnated with different alkali salts. *Energy* 2017, *118*, 1055–1065. DOI: 10.1016/j.energy.2016.10.134.

(6) Kramb, J.; DeMartini, N.; Perander, M.; Moilanen, A.; Konttinen, J. Modeling of the catalytic effects of potassium and calcium on spruce wood gasification in CO_2. *Fuel Process. Technol.* 2016, *148*, 50–59. DOI: 10.1016/j.fuproc.2016.01.031.
